# Anterolateral Thigh Flap for Acute/Primary Burn Reconstruction

**DOI:** 10.3390/ebj6020034

**Published:** 2025-06-10

**Authors:** Eva Verdaguer, Antonio Bulla, Jordi Serracanta, Danilo Rivas, Juan P. Barret

**Affiliations:** 1 Department of Plastic Surgery and Burn Center, Vall d’Hebron Barcelona Hospital Campus, Passeig de la Vall d’Hebron 119-129, 08035 Barcelona, Spain; evaverdaguerolivella@gmail.com (E.V.); antonio.bulla@vallhebron.cat (A.B.); jordi.serracanta@vallhebron.cat (J.S.); daniloantonio.rivas@vallhebron.cat (D.R.); 2 Department of Surgery, School of Medicine, Universitat Autònoma de Barcelona, 08035 Barcelona, Spain

**Keywords:** burns, burn reconstruction, microsurgery, free flap, ALT flap, flap safety

## Abstract

Introduction: The indication for a free flap in acute burn reconstruction is very specific. It should avoid several complications that are more common in the burned patient population. We propose an anterolateral thigh (ALT) flap as a first option for primary burn reconstruction in microvascular free flap reconstruction in burned patients. Patients and Methods: A retrospective review of all acutely burned patients treated with microvascular ALT free flap reconstruction between the years 2005 and 2022 in the Vall d’Hebron Barcelona Hospital Campus Burn Centre was conducted. Results: We performed 30 ALT flaps for primary burn reconstruction. The majority of patients were male (87.5%), with a mean age of 36.7 years, and 37% of patients were smokers. High-voltage electrical burns were the most common etiology. The mean time between burn injury and microsurgery was 22 days. The main recipient site was the lower limb. The flap survival rate was 96.6%. One patient required a meshed skin graft to cover a defect in the proximal third due to peripheral flap necrosis. One flap experienced mild congestion, which resolved spontaneously. Another flap had a local infection, which resolved with antibiotic therapy and surgical debridement. Conclusions: An ALT flap offers several advantages to a burned patient, provided that the surgical technique and postoperative management described in this study are followed. We propose it as the first option for primary burn reconstruction using free flaps in a burned patient.

## 1. Introduction

The primary goal of acute burn reconstruction is to achieve complete wound healing and prevent infection [1]. While most burns can be managed satisfactorily either non-surgically or by excision and split-skin grafting [2], a small proportion of patients (1.5% to 1.8% of burn patients) will require flaps to cover exposed bones, nerves, vessels, or tendons and for limb salvage [3,4,5]. Unfortunately, regional or local flaps are rarely used due to the location of most wounds in the limb or limited donor sites due to adjacent tissue damage [6,7]. Free flaps are commonly used in burn reconstruction to restore function, improve appearance, and enhance quality of life following significant tissue loss. Indications for free flap use in burn reconstruction typically include large burn wounds with deep-tissue loss extending to deeper layers, including muscles, tendons, or bones, and there is significant tissue loss that cannot be covered with simple grafts (like split-thickness or full-thickness skin grafts). Another indication for free flaps is tissue defects in functionally or aesthetically critical areas, such as the face, hands, and joints, where aesthetic outcomes and functional recovery are crucial. Free flaps can offer better contour and restore function where local flaps or grafts would be inadequate. Free flaps provide a better chance of survival and long-term healing in poorly vascularized or scarred skin, whereas certain flaps are also utilized when there is a need for sensate reconstruction, like the innervated radial forearm flap or the anterolateral thigh flap.

These indications typically require careful consideration of factors such as the patient’s overall health, available donor sites, the burn’s depth and location, and the surgeon’s expertise.

The key differences between reconstruction with free flaps in the acute phase and the late burn reconstruction phase lie in the timing, goals, and tissue conditions present at the time of surgery. Acute burn reconstruction emphasizes immediate tissue viability and coverage, often with large or robust free flaps, while late burn reconstruction focuses more on aesthetic and functional outcomes, requiring a delicate approach to address scarring, contractures, and the overall appearance of the skin.

Reconstruction and coverage with free flaps in an acutely burned patient must take into account several aspects.

A burned patient suffers from a number of systemic effects, such as post-burn hypovolemia and increased levels of inflammatory mediators, with a profound systemic inflammatory response syndrome and hypercatabolic state. Depending on the severity, this can lead to cardiovascular and respiratory instability, coagulopathy, an increased risk of infection, low tissue perfusion, and, in the worst cases, multiple organ dysfunction (MOD). Therefore, acute patients with large-total-body-surface-area (TBSA) burns and high Abbreviated Burn Severity Index (ABSI) scores require the optimization of resuscitation and critical care management. In addition, inhalation injury worsens prognosis and delays patient stability [8]. For these reasons, free tissue transfer may be delayed in many cases, and surgery should initially focus on eschar removal in burns involving a >15% TBSA [1,9]. Free flap success is dependent on an adequate blood supply and tissue oxygenation. Both the inflammatory response and the hypermetabolic state can impact vascular function, leading to increased risk of flap failure due to compromised perfusion or ischemia.

Primary/acute burn reconstruction is also dependent on the patient’s hypermetabolism and hypercoagulable state, which can lead to local complications and flap loss [7]. Vessels at the burn site are damaged by thermal and electrical burns, with disruption of the endothelium and vessel wall, leading to the development of thrombosis, aneurysms, and vascular occlusion [1], which mandates considering recipient vessels distant from the recipient site [10]. In addition, many donor sites are unavailable because of the injury itself or previous burn surgery. In the context of burns, the hypermetabolic and inflammatory states may slow down the healing process at both the donor and recipient sites, affecting tissue integration and the reconstruction’s final outcome. The inflammatory response in the donor site can increase the risk of complications like wound dehiscence, delayed healing, and infection, which can complicate the recovery of both the flap and the patient. Due to the ongoing inflammatory and hypermetabolic responses, the timing of free flap reconstruction may be delayed until the patient is more stable and the acute phase of burn injury has passed. Immediate reconstruction in the face of these responses can lead to a higher risk of flap failure. Nutritional support, fluid management, and the use of medications to modulate the inflammatory response (such as steroids or cytokine inhibitors) are often part of the management plan to mitigate the effects of these responses on free flap reconstruction. The success of free flap reconstruction in burn patients depends on managing these responses effectively through the careful timing of surgery, nutritional support, and systemic care to ensure the viability of both the donor tissue and the recipient site [1,4].

High-voltage electrical injuries deserve special attention due to the greater need for aggressive debridement, usually including affected muscles and nerves, and the vascular damage mentioned above.

In acute burn reconstruction, the choice of a free flap depends on several factors, including the location and extent of the burn injury, the patient’s overall health, and the goals of reconstruction (e.g., functional restoration or aesthetic improvement). Ideally, the free flap selected for burn reconstruction should have specific characteristics to ensure optimal outcomes. The ideal free flap should meet a number of requirements to be suitable for primary reconstruction: long pedicles are recommended due to vascular damage, and changes in the patient’s position that lead to long surgery times may be unacceptable for medical reasons. The type of operation should not increase donor site morbidity or delay rehabilitation. In addition, the donor site should not be damaged by the burn injury.

An anterolateral thigh (ALT) flap is considered the workhorse of reconstructive surgery. It fulfils all the above requirements in most cases: its pedicle length reaches distant sites, it is anatomically reliable [11,12], and it is technically easy to harvest. It is also versatile in volume and shape and can contain functional components [13]. The ALT flap provides a large, thick skin flap that is highly versatile, has a good blood supply, and is relatively easy to harvest. It can be used for covering large, deep defects, and can be adapted to various anatomical regions. The flap can also provide muscle or fascia if needed for deeper burns.

Although there are a few large case series studies on the use of free flaps in burn reconstruction, there is not much information on their use and efficacy in the reconstruction of burn patients in the acute phase. There is also still a great lack of knowledge about which is the most reliable and ideal flap for this type of reconstruction.

The aim of this study was to evaluate the results of the anterolateral femoral flap in burn patients and to validate its role in acute burn reconstruction.

## 2. Patients and Methods

We performed a retrospective review of all patient data with a descriptive analysis of all cases of primary with ALT-free tissue transfer between 2005 and 2022 at the Burns Centre of the Vall d’Hebron Barcelona Hospital. This study was approved by the Clinical Research Ethics Committee of the hospital (ID-RTF065; date of approval: 1 November 2018). A STROBE statement and checklist were utilized to improve the quality of this retrospective observational study [14].

The data collected included demographics, comorbidities, past medical history, burn type, site, and burned TBSA, recipient vessels, type of anastomosis, number of debridements prior to free flap transfer, timing of microsurgery, cause of failure, postoperative management, and outcome. The data were collected after searching the burn center patient database. Patients who had been treated with a free flap reconstruction during the acute phase of treatment were identified. From that pool of patients, those who had an ALT-free flap were selected for the final analysis. The data were then collected with full anonymization to avoid patient identification.

### 2.1. Surgical Technique

The surgical procedure was performed as described in the literature [5,10,11,12,13,15,16,17]. The preoperative identification of perforators was performed by a handheld Doppler and ultrasound examination. We only performed an Angio CT scan assessment of the donor and recipient sites in patients with pathologies such as diabetes or peripheral arterial disease.

All flaps were harvested with a subfascial approach, leaving all soft tissues intact. Subfascial dissection of the anterolateral thigh (ALT) flap is a technique used to harvest the flap while preserving the fascia and minimizing damage to the surrounding soft tissues. It is especially useful for patients who require a thicker, well-vascularized skin flap with an intact and functional fascial layer.

The ALT flap is harvested from the anterolateral aspect of the thigh and is based on the anterolateral thigh perforator artery of the descending branch of the lateral circumflex femoral artery (LCFA), which is a branch of the femoral artery. The subfascial plane refers to the layer beneath the fascia lata (the deep fascia of the thigh), and dissection in this plane involves separating the fascial layer from the underlying muscle, fat, and other structures.

The planned incision line should extend from the iliac crest down to the knee on the anterolateral thigh. The area to be harvested will depend on the defect and the desired flap size. The primary perforator vessels are usually located about 2–4 cm below the inguinal ligament and 6–8 cm medial to the anterior superior iliac spine (ASIS). As mentioned earlier, perforators may be located by either Doppler ultrasound or an angio-CT scan.

The flap is elevated in the subfascial plane by carefully separating the fascia lata from the underlying muscle and fat. This allows both the fascia and fat to be preserved, which are important for the flap’s thickness and reconstructive outcomes. Next, the perforators are identified and protected. These perforators are traced proximally to their origin in the lateral circumflex femoral artery (LCFA). The perforators are dissected gently to avoid unnecessary trauma and ensure they are left intact to maintain adequate perfusion to the flap. Once the perforators are isolated and the subfascial plane is adequately dissected, the flap can be elevated. This involves lifting the skin, fat, fascia, and the perforators, ensuring the flap’s full vascular supply is intact. The flap is then mobilized from its surrounding tissue while maintaining its connection to the perforators. The harvested flap is transferred to the recipient site, where it can be inset and sutured in place, and the vessels anastomosed.

### 2.2. Postoperative Management

All patients received daily antiplatelet therapy (100 mg of aspirin) for two weeks and DVT prophylaxis (40 mg of low-molecular-weight heparin, subcutaneously) while bedridden. The rest of the general management was carried out according to our burn care protocols through a multidisciplinary team approach. For lower limb reconstruction, absolute rest with limb elevation for at least ten days was mandatory.

The monitoring of the flaps was performed by means of periodic clinical inspection and a hand-held portable Doppler. In the last two years of the series, monitoring was performed by the former plus INVOS^®^ technology (Medtronic Iberica, Madrid, Spain) The monitoring system provides continuous, non-invasive indications of changes in the regional blood oxygen saturation (rSO2) of somatic tissues.

## 3. Results

From January 2005 to December 2022, we performed 52 microsurgical interventions in acutely burned patients. The ALT free flap was the most frequently used microvascular flap for soft-tissue coverage, embracing 57.7% of the overall flaps (30 out of 52 flaps). The ALT flap was followed by latissimus dorsi and superficial circumflex iliac artery perforator (SCIP) free flaps. The results of all types of flaps utilized are summarized in Table 1.

Most patients treated with ALT flaps were male (87.5%), with a mean age of 36.7 years (ranging from 16 to 71 years). Smoking habits were not recorded in 7 patients; 6 patients were active smokers (20%), and 17 were non-smokers. One patient had cardiovascular risk factors (diabetes and dyslipidemia).

Severe electrical burns were the main reason for microsurgical reconstruction (17 out of 30 patients), followed by flame burns (8 out of 30) and contact burns (5 out of 30). In most patients (75%), the estimated burn area was <15% TBSA.

The time between burn injury and microsurgery ranged from 3 to 60 days, with a mean of 22 days. The average number of debridements prior to flap transfer was 1.3 and depended on the assessment of the wound by the primary surgeon and the need for further debridement.

**Figure 1:**
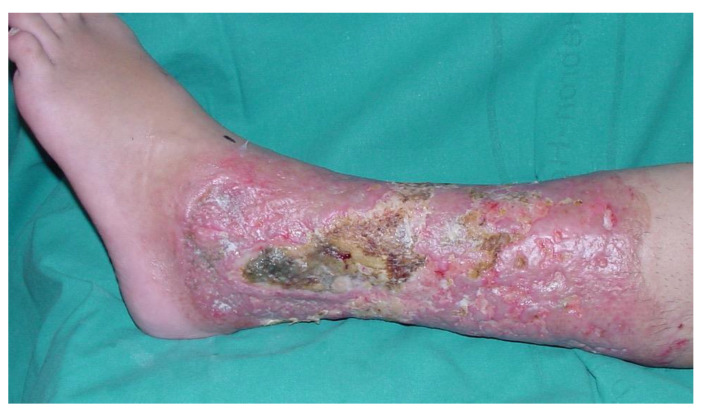
Exposed lateral malleolus after 6% full-thickness contact burn.

**Figure 2:**
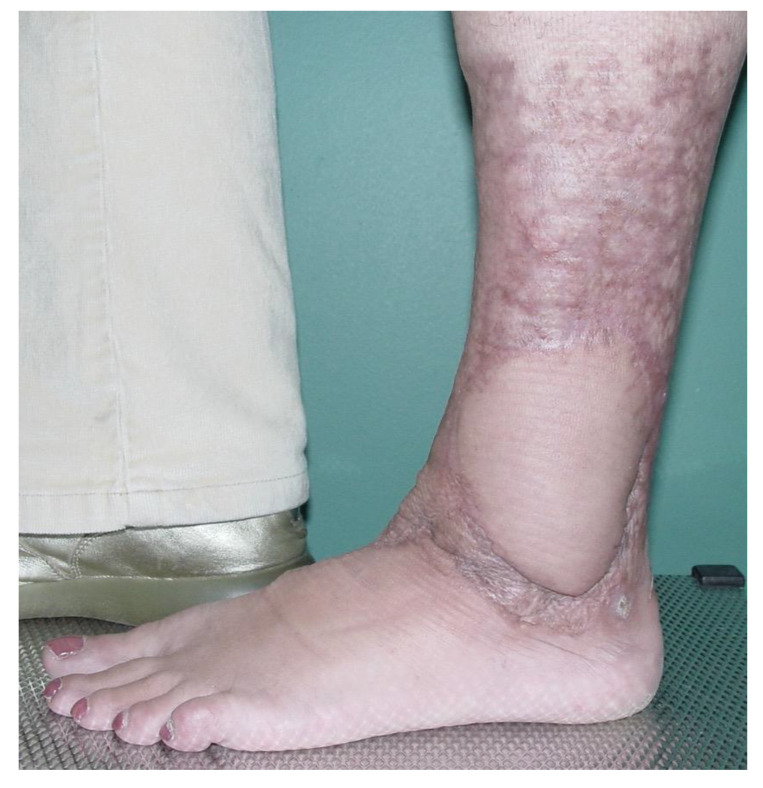
Same wound after acute burn reconstruction with an ALT free flap.

The main recipient site was the lower limb (25 cases out of 30, mostly in the foot and ankle (21 flaps); see Figure 1 and Figure 2). Other anatomical areas covered by ALT flaps were the legs, thighs, hands, forearms, and scalp. Two cases included a functional component by adding vascularized fascia lata to reconstruct the Achilles tendon [14]. The results for the ALT flaps are summarized in Table 2.

Most anastomoses consisted of one artery and one vein (73% of flaps). In 86% of the cases, the anastomosis was end-to-end, and only in 14% of the cases, the arterial anastomosis was end-to-lateral because the leg and/or foot vascularization was dependent on an arterial trunk.

All flaps but one survived (96.6% survival rate). One patient suffered partial flap necrosis in the proximal third and required a subsequent meshed skin graft. One flap had mild congestion, which resolved spontaneously. Another flap presented with local infection, which was resolved with antibiotic therapy and surgical debridement. The flap that did not survive suffered a venous thrombosis that could not be recovered. Venous anastomosis was performed with a coupler. No donor-site complications were reported. No flap complications were reported in the long-term follow-up.

## 4. Discussion

In a series of 30 consecutive burned patients, an ALT free flap with a subfascial dissection proved to be a reliable technique to reconstruct complex defects in acute burn reconstruction. All flaps survived, and only minor wound problems presented in two patients.

The ALT flap technique has a number of characteristics that make it suitable and convenient for the primary reconstruction of burn wounds. It adapts to the needs of the burned patient, provides soft coverage and functional reconstruction when needed, and is safe and reliable. Dissection is straightforward in most cases due to its highly consistent anatomy, and it is harvested in the supine decubitus position, reducing the anesthesia time and remote complications during surgery. Its size can be adapted to the patient’s wound size [12].

A higher rate of free flap loss has been described in acute burns in the literature when compared with other flap transfer series, such as traumatic or oncological ones [16,17,18]. According to a systematic review of the literature [1], most cases are due to arterial or venous thrombosis. The suggested reasons include incomplete debridement and a higher rate of local infection [10,15,19,20], damaged vessels in thermal and electrical injury [21], and systemic burn effects related to SIRS and hypermetabolic response. Despite being indicated in patients at high risk of flap failure, such as smokers, patients with vascular risk factors, and atherosclerosis, all free flaps survived in our series.

There may be a number of reasons for complete flap survival in our study. The harvesting approach was always subfascial, which increased vascularization and improved survival [22]. ALT flaps were only trimmed during surgery if they were too bulky. Another reason may be that distant anastomosis was performed in all cases, at the point where the recipient vessels were assessed by the surgeon and considered to be in good condition, at a minimum of 3 cm from the injured area [23]. The ability to reach a long pedicle makes the ALT flap useful for placing the anastomosis away from the injured area.

According to the literature, the timing of reconstruction is also considered a possible cause of flap failure, although there is no clear evidence of the best time for surgery [1,5,21,24]. An important limitation of immediate free flap transfer is patient instability. In addition, it has been suggested that the relationship between the timing and failure rate may be confounded by incomplete debridement [21]. We believe that complete debridement is an essential step for successful reconstruction to ensure the viability of the remaining tissue and to prevent local infection. We performed as many debridements as necessary to achieve a clean, non-infected wound bed. Great care must be taken with the debridement of electrical burns as the injury evolves over time. For this reason, we performed serial debridement until we were completely confident that the area was clean and free of devitalized tissue. In our review, 16 of the 30 patients had one or more debridements prior to the final free flap reconstruction.

In our case series, the majority of anastomoses consisted of one artery and one vein (73% of procedures). Nevertheless, whenever possible, we anastomosed two veins to avoid congestion and to achieve a more anatomical reconstruction, although some of the literature advocates for only one venous anastomosis [24,25].

Much attention must be paid to postoperative management. The main aim in the weeks following flap transfer was to prevent flap congestion and limb oedema. The flaps were continuously assessed for their response to gravity and ambulation. A delay in these activities was indicated by the patient’s and flap’s response to the rehabilitation program, if necessary.

One of the disadvantages of the ALT flap is the thickness of the cutaneous palette, which is highly variable between patients. This is an aesthetic issue, which is not an important goal in primary reconstruction. The flap can be thinned by liposuction or lipectomy in subsequent procedures. In areas under pressure, such as the first metacarpal-phalangeal area, the full thickness must be maintained to avoid future wounds.

A possible contraindication to performing an ALT flap is the presence of femoro-popliteal obstructive arterial disease in the donor thigh, in which case the descending branch of the lateral circumflex femoral artery plays a key role in supplying blood to the distal popliteal artery [1].

Otherwise, the morbidity of the ALT donor site is very low, and the use of pre-sutures or regional flaps for direct closure allows the avoidance of grafts and aesthetic disadvantages when necessary.

## 5. Conclusions

The ALT flap with subfascial elevation allows distant-vessel anastomosis and a high survival rate in acute burn reconstruction. Serial debridement is recommended to achieve a clean and non-infected wound prior to flap transfer. The reconstruction of acute burns with ALT flaps offers advantages to the burned patient in terms of safety and versatility, with little or no complications and morbidity.

The subfascial dissection of an ALT flap is a specialized technique that allows for the harvest of a robust, well-vascularized flap with intact fat and fascia, making it ideal for reconstructing large, deep burn wounds while minimizing muscle disruption. The procedure demands careful planning, precise dissection to preserve the perforators, and thorough postoperative monitoring to ensure the success of the flap. It provides a reliable option for burn reconstruction, especially in areas requiring thick, pliable tissue.

## Figures and Tables

**Table 1:** Types of flaps and types of defects.

Flap	Number of Procedures	Percentage of Flap Survival
Anterolateral thigh (ALT)	30	97%
Latissimus dorsi (LD)	9	
Superficial circumflex iliac artery perforator (SCIP)	7	71.5%
Thoraco-dorsal artery perforator (TDAP)	2	77.8%
Gracilis	1	100%
Radial forearm (RF)	1	100%
Superficial temporal fascia	1	100%
Pure skin perforator from inguinal area (PSP)	1	100%

**Table 2:** ALT flap characteristics.

	Number of Flaps and/or Percentage
Location of injury	Lower limb (25), wrist (3), and scalp (2)
Type of injury	Electrical (17), flame (8), and contact (5)
Partial flap necrosis (margin or tip)	1
Infection	1
Total flap necrosis	1 (venous thrombosis)

## Data Availability

The data presented in this study are available upon request from the corresponding author due to the ethics of our IRB.
